# Conduct of cancer clinical trials: a qualitative study reporting views of patients, caregivers, public and clinical researchers

**DOI:** 10.3332/ecancer.2025.1950

**Published:** 2025-07-22

**Authors:** Soumitra S Datta, Bidisha Samanta, Sanjoy Chatterjee, Indranil Mallick, Atul Sharma, Gargi Gangopadhyay, Shreshta Chattopadhyay, Chitralekha Bhowmick, Neha Chawla, Caroline Clarke, Duncan Gilbert, Usha Menon

**Affiliations:** 1 Department of Palliative Care and Psycho-Oncology, Tata Medical Centre, Kolkata 700160, India; 2 MRC Clinical Trials Unit, Institute of Clinical Trials and Methodology, University College London, 90 High Holborn, London WC1V 6LJ, UK; 3 Department of Radiation Oncology, Tata Medical Center, Kolkata 700160, India; 4 Department of Medical Oncology, All India Institute of Medical Sciences, New Delhi 110029, India; 5 Department of English, Ramkrishna Sarada Mission Vivekananda Vidyabhavan, 33, Sri Maa Sarada Sarani, South Dum Dum, Kolkata 700055, India; 6 Translational Health Science and Technology Institute, NCR Biotech Science Cluster, 3rd Milestone, Faridabad – Gurugram Expressway, Faridabad, Haryana 121001, India; 7 Research Department of Primary Care and Population Health, University College London, London WC1E 6BT, UK

**Keywords:** patient and public involvement (PPI), clinical trials, cancer, oncology, consent, LMICs

## Abstract

**Background:**

The rising cancer burden in low- and middle-income countries (LMICs) has been accompanied by an increase in clinical trials. However, there is a paucity of research from LMICs on patient preferences for trial participation.

**Methods:**

We undertook a cross-sectional qualitative study using in-depth interviewing to explore the views of Indian cancer patients (*n* = 11), caregivers(*n* = 10) and public (*n* = 10), regarding clinical trials. Clinical researchers (*n* = 10) were also interviewed. Data were analysed using the framework of qualitative content analysis.

**Results:**

Five themes were identified regarding clinical trials: a) ***Perception*:** Only a minority had a prior understanding; when explained, most were willing to be randomised and attend additional monitoring visits. b) ***Recruitment*:** Consensus that trial discussions should be with the patient, with caregivers and family included where appropriate, variability in when a patient should be first approached. c) *Patient ****information*:** Need for both written and audio-visual information material using simple local language. d) ***Benefits/adverse effects***: Discussion of all pros and cons, including the possibility of dying was preferred. There were divided views regarding disclosure of all versus common risks. Challenges in understanding quantitative risks/benefits were voiced. e) ***Consent:*** Honesty and transparency, imbalance of power/trust between trialists and participants and financial vulnerability of patients were voiced by participants.

**Conclusion:**

Cancer clinical trials in LMICs can be enriched by patient and public involvement during planning research and conduct of the clinical trial. The financial vulnerability of patients and the power imbalance between them and researchers need to be addressed, especially in international multiregional clinical trials.

## Introduction

Cancer burden is growing rapidly in low- and middle-income countries (LMICs) [1, 2]. However, the majority of current cancer research is conducted in high-income countries [3]. There have been calls to rebalance the global inequity of research priorities and settings [4, 5]. Roy and Mathew [6] reported that disproportionately low attention was paid to cancers that have a high disease burden in India in an audit of 350 cancer clinical trials recruited from India between 2007 and 2017. A related but separate issue is that recruitment to clinical trials in LMICs follows international guidelines that have been mostly developed in the west. Tinto *et al* [7] have pointed out the challenges of applying ethical models developed in Western countries to resource-limited settings due to the disparity in the nature of care patients participating in clinical trials are able to access compared to those accessing routine care.

With increased awareness about patient rights and democratising of health, ‘patient and public involvement (PPI)’ in clinical research has been formalised in many high-income countries [8]. There is a paucity of research on patient preferences around trial participation from LMICs [9]. A systematic review on patients’ understanding of informed consent, reported that patients have a variable understanding of ‘the purpose of the study’, ‘adverse events’, ‘benefits of participation’, ‘placebo’, randomisation and ‘freedom to withdraw participation’ [10]. One way to bridge this gap is to explore the views of potential patients during the planning phase of clinical trials. The positive effects are likely to be bidirectional [11] While this is increasingly a requirement in high-income countries, it is not the norm in LMICs. We report on a study from India that explores the views of cancer patients, caregivers and the lay public alongside trial staff on design, conduct, recruitment and retention in cancer trials.

## Methods

### Research design

This study is a cross-sectional qualitative research study using in-depth interviews of patients, caregivers and the lay public in India as well as clinical researchers involved in running cancer clinical trials. The goal was to explore the perceptions and views of cancer patients, their caregivers and members of the public regarding cancer clinical trials. Ethics approval was obtained from Tata Medical Center Ethics Committee, Kolkata (2020/Govt/35/IRB42). All the participants provided written informed consent. GRIPP-2 guidelines [12] for the conduct and reporting of studies on PPI in research and COREQ guidelines for qualitative research [13] were followed.

### Setting

The study was conducted in a specialised oncology hospital predominantly catering to patients across a wide socio-economic spectrum from eastern India and neighbouring countries. The hospital offers subsidised treatment to many patients.

### Study team

The study team consisted of a consultant psychiatrist, three clinical psychologists, two consultant radiation oncologists, two clinical oncologists, one gynaecological oncologist, one medical oncologist and one health economist. There was a mix of expertise and skills amongst the research team consisting of members who had expertise in conducting large clinical trials and as well as others in conducting qualitative research studies.

### Engagement of patient–partners

Patients and caregivers were consulted initially at the time of designing the study and at the time of choosing the topics and areas to be explored. One of the co-authors of the study was the main caregiver for a family member with cancer and has supported several cancer patients who needed logistical support mobilising a large social network.

### Recruitment of patient, caregivers and public

#### Inclusion criteria

Patients: Adult cancer patients of any gender who were fluent in spoken Bengali or English with a known diagnosis of cancer were recruited based on a purposive sampling so that all common cancer is represented.

Caregivers: Adult caregivers of patients with cancer who were fluent in spoken Bengali or English, who looked after a cancer patient and made important treatment-related decisions were interviewed to get their views.

Public: Members of the public who were aware of the cancer care pathway in India, mostly through their role in the civic society. They included managers, patient advocates and advisors to cancer charities, supporting treatment and/or research in cancer in India.

#### Exclusion criteria

Patients and caregivers were excluded if their treating clinician perceived them to be emotionally vulnerable and potentially upset by the issues raised during the interviews.

#### Recruitment

Patients and caregivers were recruited from those attending out-patient clinics at the hospital. The study followed a stratified purposive sampling. Patients and their caregivers with all the common cancer types as reported in the Global Cancer Observatory (GLOBOCAN) 2022 for India were included in the study [14]. Participants were purposively selected such that their views represented a wide range of backgrounds in terms of age, gender, sociodemographic status and a particular type of cancer diagnosis. The members of the public, invited to participate in the study, were from the same catchment area (eastern India) as the patients and caregivers. Efforts were made to ensure that members of the public represented a diverse range of age, gender and professional backgrounds. Health professionals were those directly involved in the conduct of cancer clinical trials at the hospital. They included cancer clinicians who had experience as principal investigators for cancer clinical trials, trial coordinators and ethics committee members.

#### Data collection method

The socio-demographic data of respondents were collected using a predetermined proforma. In-depth interviews were conducted to collect qualitative data. The interviews were conducted by an experienced qualitative researcher (SD) familiar with the landscape of clinical trials and with handling difficult conversations. Interviews were audio-recorded and transcribed verbatim. SD was not part of the clinical team that treated participants or the clinical trials unit where the recruited professionals worked. This ensured that the interviewees could open up and freely give their opinions on any topic that they felt was relevant to the research questions being explored.

#### Data processing and data analysis

The data analysis was jointly done by the research interviewer (SSD) and a psychologist (BS). The entire data were charted by the researchers involved in the data analysis. A qualitative content analysis framework was used for data analysis [15] as it is particularly suitable for exploratory research with a large volume of data, and for studying sensitive phenomena. Two of the researchers (SSD and BS) built a coding frame as suggested by Schreier [16] by following the steps of a) selecting, structuring and generating categories; b) defining categories and c) modifying and explaining the frame. The data were analysed using NVivo 12 (QSR International) software. Interpretation of the data by SSD and BS was done by adapting an ‘empathic stand’ while remaining as close to the data as possible.

#### Results

The study involved 31 participants (11 patients, 10 caregivers and 10 members of the public) who were interviewed during April–Sept 2023 at a tertiary cancer centre in east India (Table 1). The patients were balanced for gender, but there was an over-representation of women among caregivers. The majority of the female patients were housewives. Patients included those with the most common LMIC cancers – breast, gynaecological, genitourinary, lung and head and neck. The majority (60%–100%) of the participants had completed higher education. The members of the public were from a wide range of professional backgrounds. The ten research staff interviewed included clinicians, nurses and clinical research staff (Table 1).

### Patients, caregivers and lay public

**Figure 1.**
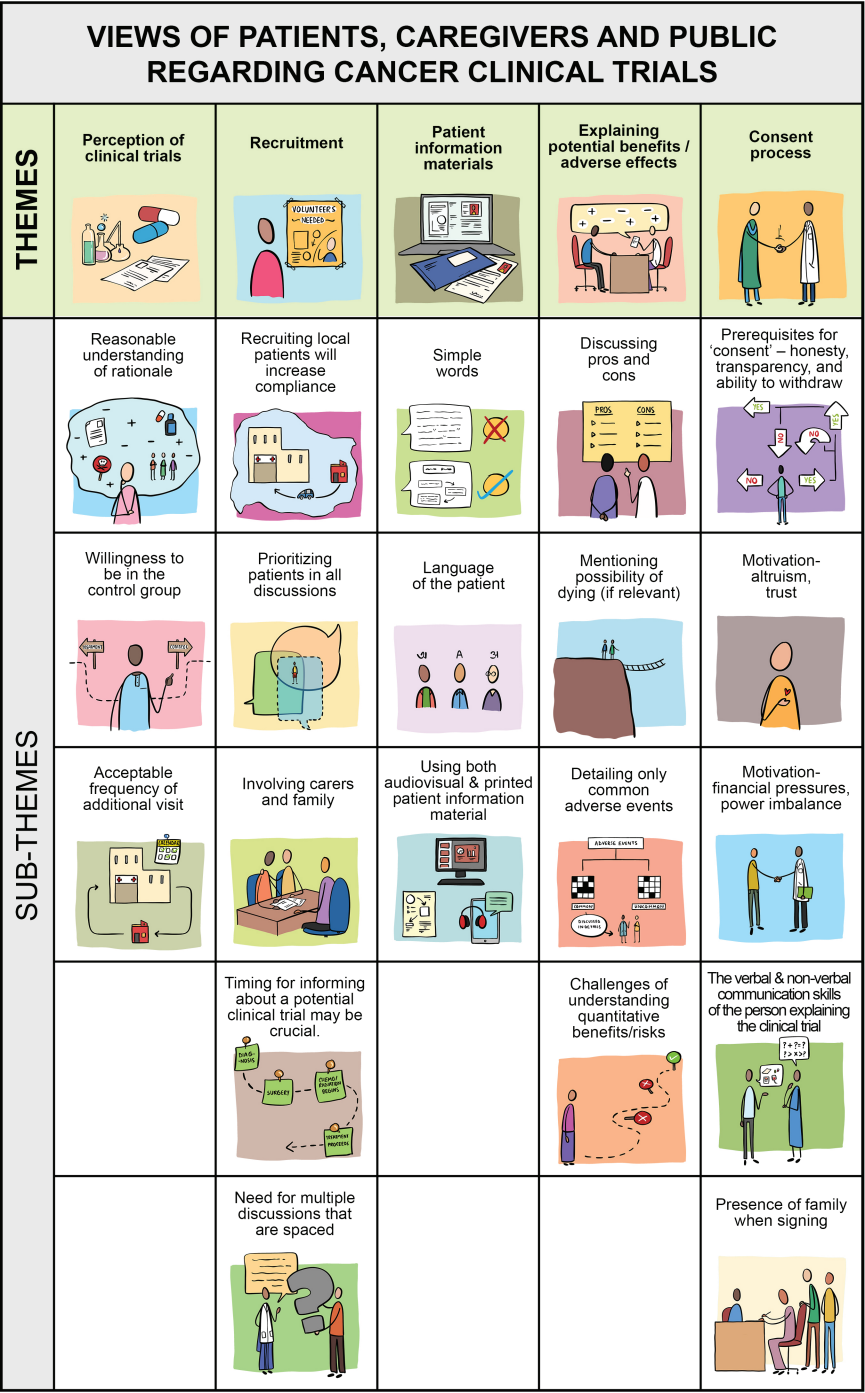
Pictorial coding tree of the views of patients, caregivers and public.

The following themes emerged on analysis of their interviews (Table 2, Figure 1, Video 1):

**Theme 1: Perception of clinical trials:** A minority of patients, caregivers and lay public had some understanding of clinical trials. Three of the eleven patients (3/11, 27%) and four of the caregivers (4/10, 40%) had heard of the term ‘clinical trial’ prior to our interview. One patient mentioned that ‘my son and daughter-in-law participated in a Covid vaccine trial’ (Patient 10). Given the uncertainty regarding outcomes, an elderly patient suggested that his preference was for older individuals to be approached for cancer treatment trials, ‘Today, if I die, it will not harm anyone. But if an 18-year-old boy or girl dies, it hurts much more’ (Patient 11). A different view was expressed by a third patient – ‘I think I would participate in a trial even if there was the slightest chance that it might help me in the long run and despite knowing that I may not be in the intervention arm.’ (Patient 4). A caregiver voiced that ‘If the patients perceive that by participating in the trial there is increased chances of recovery, then they would participate even if there are some risks’ (Caregiver 3). Another caregiver said, ‘I wouldn’t have any objections to clinical trials because of the century we are in, the new diseases, new epidemics, new pandemics such as Ebola, Covid and so on. Clinical trials are a way forward to be prepared’ (Caregiver 5). A member of the public pointed out that he would be willing to participate in a clinical trial to allow the trialists ‘to test the results of a drug, the bad effects and the good effects’ (Public 1). He went on to elaborate that ‘I really would not mind even if I was part of the control group if the trial reaches its desired end’. A member of the public commented on the acceptable frequency of visits – ‘If it is once a month, I think, patients will come for the trial-specific treatment because it is a new way of treating and may be beneficial for the patient’ (Public 4).

**Theme 2: Recruitment to a clinical trial:** In LMIC settings, patients often travel long distances to access treatment. Keeping this in context, a caregiver suggested – ‘I think it is always better to recruit patients from the same city. It’s about the health of the patient, they can’t always come, and they might lose interest. At least if it is in the same city and you are compensating for the travel, then most people would still think that it is okay, I’ll go and get it checked (if there is an adverse effect)’ (Caregiver 7).

While all agreed that the patient and caregivers should be part of the discussion, there was a difference of opinion as to the order in which they should be approached – ‘I feel that priority must always be given to the patient. I would want my family to understand it, but I should first understand what I am about to go through’ (Patient 4); ‘I think first the patient and if the patient is convinced it is fine, then consult the family’ (Patient 9); ‘Both patient and family members should be informed together’ (Patient 5); ‘If we only tell the patient, the patient may agree but not the patient’s family. So, I think it’s better to take the permission of the family members first’ (Patient 10).

There was also variability in what patients felt might be optimal timing for initial discussions about a clinical trial – ‘Even if the trial starts recruitment following completion of standard treatment, I think approaching at the start of treatment will instill hope’ (Patient 2); ‘I would prefer it during my treatment when I'm really focused on getting well and on getting cured’ (Patient 4). The need for multiple discussions was mentioned by several respondents ‘It shouldn’t be left to only one meeting. It takes a number of discussions before you begin to understand. You have to be very understanding towards the patient’s needs’ (Patient 9).

**Theme 3: Preference for type of patient information material related to a trial:** The need for simple sentences was reiterated, together with an emphasis on the use of local language – ‘I think break it down, in a language that is accessible to the person concerned and using local dialect as much as possible’ (Public 8). The value of both audio-visual information material and printed/written material were emphasised by respondents – ‘I would say that a combination of both (leaflet and video) probably helps because once you go through the audio visual, you get a bit of an idea and then people generally tend to forget about it. The leaflet would be there as a reminder of what we have seen and be a synopsis of the entire thing. I think a combination of both probably will work better’ (Public 3).

**Theme 4: Explaining potential benefits/adverse effects:** The patients highlighted the need for absolute transparency – ‘I need to be explained the pros and cons’ (Patient 4); ‘I think patients should be told all the side effects. The patient should know; they have a right to know’ (Patient 5) and so did the caregivers – ‘I think it is necessary for the patient to know everything’ (Caregiver 7). This includes the chance of dying as a result of trial participation – ‘Yes, absolutely, patients should definitely be informed about the possibility of death.’ (Patient 5), ‘This is a bit tough. Those who are strong-minded will say that it’s ok. What can happen? If not today, tomorrow I will die’ (Patient 11); ‘Ethically you should inform the patient about the possibility of death, but they will be very scared to participate in the trial’ (Caregiver 10).

On the other hand, there was a view that only the common side effects should be discussed – ‘The patient should be told just the common side effects, because if you tell them too many details, they will not agree. They will also not understand. It is important for them to know that there are some side effects involved. Just mention the common side effects and see how they react’ (Caregiver 10).

The difficulty in understanding potential benefits or risks when it was presented as numbers and percentages – ‘I don’t see the benefit as measurable. I do not measure it on a quantitative scale. I don’t think people try to measure this’ (Patient 5). Along similar lines, a caregiver mentioned that the absolute value of potential improvement even if minimal was irrelevant, ‘To me if it is 6% or 5% or 2% additional benefit I would like to participate’ (Caregiver 4).

**Theme 5: Providing consent:** A caregiver emphasised that ‘The ‘right to information’ and ‘transparency’ and the ‘right to back out’ is crucial. If they agree and later think that ‘this is not actually going well’, then they should not be coerced to continue’ (Caregiver 7). Trust and imbalance of power between health professionals and patients were mentioned as a barrier to a free and fair discussion during the consenting process – ‘In this society, where there is inequity of power, doctors, more often than not, are akin to God. If the patient relies on the doctor, he will consent irrespective of knowing about the consequences because the belief is that ‘doctors cannot do anything wrong, and he will do what is good for you’ (Public 5). An altruistic basis for the motivation to participate in a trial was also elicited – ‘When you hear of a medical trial being done, you are doing it more for the greater good and for the future advancement of medicine and people would want to be part of something like that to benefit future generations.’ (Caregiver 8). Another respondent discussed the role of communication skills and empathy – ‘Who is telling matters a lot. The body language of the person who is saying it also has an effect. If they are told in a caring way, then they will start thinking. Consent may take time, but it should make them think. If people are made to understand nicely then they will give consent’ (Public 6). A caregiver mentioned about the role of the family members in LMICs during the consenting process – ‘It is not possible to give consent on one’s own …consent can be given only after discussing the opinion of everyone.’ (Caregiver 2).

### Clinical research staff

Researchers involved in conducting clinical trials expressed their views and reflected on their experiences. One of them mentioned the advantages of introducing a clinical trial initially through a trial information leaflet – ‘I think it is a better practice to give that person something to read. It can be the information leaflet with the consent form that provides information on the trial. Give them time to read and come back to you with questions. Then you answer those questions and have a more meaningful conversation’ (Health professional 1). A trial coordinator reflected on the difficulties of having a meaningful discussion with a cancer patient given the cost of treatment, the need for most to personally cover the majority of the cost and the fact that a significant proportion are from a financially deprived background – ‘The larger proportion of patients carry a huge financial burden and are quite helpless, which is why they agree on the possibility of any sort of treatment that can happen free of cost. As per my experience, when we approach them, they often do not think of any negative consequences. They would agree to any free treatment that may have potential benefits, regardless of the side effects’ (Health professional 4). A researcher echoed this concern about the difficulties of having a fully informed discussion regarding safety and potential adverse effects in the face of financial deprivation and emotional vulnerability – ‘As a non-clinician, sitting through those consenting sessions, I have felt that percentages don’t register in your mind because you need the treatment, and you don’t have money and you are feeling helpless. Here is this trial which is giving you the drug that is very costly otherwise. Therefore, their focus is ‘give me this treatment that I can't afford’ or ‘I am not getting this treatment anywhere else’. They are unable to assimilate the safety aspects of participation’ (Health professional 1). A trial coordinator also mentioned the advantages of having a family member during the consenting process when interacting with some of the less educated patients – ‘It can get problematic whenever we need to get a document signed by someone who is uneducated and from a financially under-privileged background. They often get scared when they are asked to sign something. That is the reason we want a family to be present at the time of taking consent, especially a male member like a husband or a son, in case of aged patients. The patient feels reassured when a family member is there with them. Even when the patient does not understand all the intricacies of the trial, the family members could grasp what is going on, and would in turn help the patient comprehend’ (Health professional 4).

## Discussion

This qualitative study of cancer patients, their caregivers and the lay public found that though only a minority had a good understanding of clinical trials, there was consensus that the patient should be part of all trial-related discussions, with caregivers and family included where appropriate. The need for transparency, with risks and benefits explained using simple sentences in the local language was highlighted. Trust and imbalance of power between patients and health professionals were raised as a barrier to a free and fair discussion about the pros and cons of trial participation. The latter was reiterated by the clinical researchers interviewed, who commented on the vulnerability of patients who cannot afford cancer treatment. The need to address this power imbalance is especially relevant to international multiregional clinical trials where new cancer drugs are investigated across a variety of healthcare settings. The vulnerability related to financial deprivation applies to patients from both LMICS as well as uninsured patients from high-income countries.

Our study adds to the identified need for the voices of patients and the public to be included in LMIC research studies [9]. In a recent global scoping review on PPI in cancer research, only 3% (2/66) of included studies were from LMICs [14]. This contrasts with high-income countries where there is increasing involvement of patient and public representatives in all stages of clinical trials from funding decisions to design to interpretation of results. A recent systematic review has identified clear benefits of accommodating patients' views on enrolment and retention in clinical trials [16].

The role of financial vulnerability that was highlighted by our participants was also reported in a previous study from India which found that patients, especially those with financial constraints, were more likely to enroll in a trial where treatment was free [17]. This is compounded by the power imbalance at the interface of the doctor-patient relationship [18]. Recent efforts to address the latter, have included inclusive learning models called ‘dialogue cafes’ that provide opportunities for the exchange of ideas and perspectives between patients and professionals [19] and co-designing of personalised care plans in oncology [20]. Creative methods have also been suggested to communicate complex statistical results of clinical trials [21].

The inclusion of family/caregivers in discussions about trial participation is in keeping with our previous finding [22] that patients want them to be part of clinical decision making. This is also common clinical practice in LMICs. However, the importance of including family/caregivers in trial-related discussions finds little mention in the ICH (E6) GCP guidelines [23] where the emphasis is only on the subject or the subject’s legal representative. As a result, there is little discussion of this aspect during GCP training.

The study has several strengths: a) robust design and implementation using COREC [13] and GRIPP-2 guidelines [12], b) multidisciplinary team with expertise in cancer, psychology, clinical trials and qualitative research c) PPI representative on the research team d) wide participation ensuring inclusion of multiple views from patients, caregivers, members of the public and clinical researchers, e) inclusion of patients from all stages of the cancer journey – at diagnosis, during and after treatment. The limitations include cross-sectional design and inclusion of one centre. However, it needs to be noted that the tertiary cancer centre serves a large population with patients and their caregivers hailing from eastern India and neighbouring countries.

We found that although only a minority of patients and caregivers in LMICs may have a good prior understanding of a clinical trial, once the concept is explained, they can give valuable suggestions on various aspects of the trial, thus corroborating our hypothesis that studies in LMICs should be including PPI collaborators, as is currently done in the west. The poor prognosis coupled with the cost of routine cancer care magnifies the power imbalance between researchers and participants enrolling in clinical trials of new cancer drugs. Clinical trialists therefore need to take extra steps to ensure that patients weigh pros and cons before participation.

## Conflict of Interest

The authors declared they have no conflict of interest

## Figures and Tables

**Video 1.**
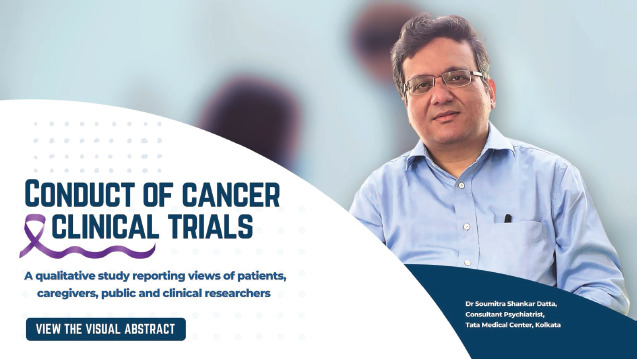
The themes that emerged from the research on PPI in cancer clinical trials are presented in the video abstract. To view the video, click here: https://vimeo.com/1101492270/4897d7ee42?share=copy.

**Table 1.** Demographic characteristics of respondents.

Variable	Patients *n* = 11 (%)	Caregivers *n* = 10 (%)	Public *n* = 10 (%)	Professionals *n* = 10 (%)
Gender				
°Male	5 (54.54)	3 (30)	6 (60)	8 (80)
°Female	6 (45.45)	7 (70)	4 (40)	2 (20)
Age range				
°40–49	3 (27.2)7	2 (20)	1 (10)	6 (60)
°50–59	4 (36.36)	1 (10)	7 (70)	4 (40)
°60–69	1 (9.09)	7 (70)	0	0
°70–79	3 (27.27)	0 (00)	2 (20)	0
Education level attained				
°Primary	1 (9.09)			
°Secondary	4 (36.36)			
°Graduation	5 (54.54)	7 (70)	2 (20)	3 (30)
°Post-graduation (including PhD)	1 (9.09)	3 (30)	8 (80)	7 (70)
Occupation				
°Manager	0	1 (10)	0	0
°Professional	2 (18.2)	1 (10)	4 (40)	0
°Technician and associated professionals	0	1 (10)	3 (30)	1 (10)
°Clerical support worker	0	2 (20)	2 (20)	1 (10)
°Skilled agricultural workers	1 (9.1)	0	0	0
°Student	0	1 (10)	0	0
°Home maker	5 (45.4)	3 (30)	0	0
°Researcher	0	0	0	4 (40)
°Retired	2 (18.2)	2 (20)	1 (10)	0
°Health professional	1 (19.1)	0		4 (40)
Cancer (index patient)				
°Breast cancer	1 (9.09)	1 (10)		
°Gynecological cancers	4 (36.36)	4 (40)		
°Head and neck cancers	1 (9.09)	2 (20)		
°Gastrointestinal cancers	2 (18.18)	3 (30)		
°Genitourinary cancers	1 (9.09)	0		
°Lung cancer	2 (18.18)			
Relation of primary caregiver with cancer patient				
°Son		2 (20)		
°Daughter		3 (30)		
°Wife		2 (20)		
°Caregiver		1 (10)		
°Sister		1 (10)		
°Daughter- in- law		1 (10)		

**Table 2.** Summary of themes, key findings and quotes from patients, carers and public.

Theme / finding	Quotes
**Perception of clinical trials**	
Reasonable understanding of rationale	“It is a work in progress and therefore if I am part of a clinical trial, so they will use my system, my physical system as a way to test results of how that pill is exactly working, the bad effects and the good effects” (Public 1) “I don’t think it can be explained and everyone would understand. For people from rural areas, it will still be considered as a ‘treatment’. They may think ‘there is a new treatment that nobody has tried, and I might get better with it’.” (Public 9)
Willingness to be in the control group	“When you know you are not getting the benefit out of it maybe you might think of not coming.” (Carer 8) “I really would not mind, if I am part of a trial and if I happen to belong to the group that is given a placebo and I come out of the trial ‘as I was’ and if the trial reaches its desired end, it's okay. I will not have a problem with that.” (Public 1)
Acceptable frequency of additional visit	“If it is once a month, I think, patients will come for the trial specific treatment because it is a new way of treating and may be beneficial for the patient.” (Public 4)
**Recruitment to clinical trials**	
Recruiting local patients will increase compliance	“I think it is always better to have it in the same city. It’s also about the health of the patient, they can’t always come, and they might lose interest. At least if it is in the same city and you are compensating for the travel then most people would still think that it is okay, I’ll go and get it checked.” (Carer 7)
Prioritizing patients in all discussions	“I feel that priority must always be given to the patient. I would want my family to understand it, but I should first understand what I am about to go through.” (Patient 4) “I think first the patient and if the patient is convinced it is fine, then consult the family and bring the family.” (Patient 9) “Sometimes there are family members with whom you cannot discuss anything. The patient is much more intelligent and understandable than family.” (Patient 11) “I think it is necessary for the patient’s side to know everything. One because, the effects are on them. Also, on moral grounds, they should know what they are getting into. If they decide to go for it, then it's very good and if they decide that they don’t want to risk it, then so be it.” (Carer 7)
Involving carers and family	“If we only tell the patient, the patient may agree but not the patient’s family. If the patient doesn’t agree, family members may agree to it. So, I think it’s better to take the permission of the family members first.” (Patient 10). “Both patient and family members should be informed together.” (Patient 5)” “Maybe first caregiver should be brought into the loop and they can take a call how much they want to tell the patient.” (Carer 8) ”If the family members are giving the go ahead with the consent and they are not being explained in detail of the side effects and if they get to know after 1–2 months this is what happened because of this clinical trial, I don’t think it would leave the family members in a good positive mind.” (Carer 5).
Timing for informing about a potential clinical trial may be crucial*	“I think approaching right at the beginning will instill hope.” (Patient 2) “If I am talking from a patient’s perspective, I would prefer it during my treatment when I'm really focused on getting well, on getting cured of my cancer. Doctor can put in a word and say once this treatment is completed there is another trial you could go into which will help you further in stopping the reoccurrence of this. Not at the ‘end of the treatment’ - that is my take.” (Patient 4) “At the end of the miserable radiotherapy and chemotherapy, that I have gone through, I get this good news that, “hey, it’s worked. You got 5 years. I told you this earlier. You may want to try this; you might get another 5 years.” (Patient 9)” “Probably one could start after surgery”. If the surgery is successful and they would have established a mutual trust with the doctor and the medical team and with the hospital. So, once that part is over and the patient is on his or her way to recovery, then he's more open-minded. (Public 3)
Need for multiple discussions that are spaced	“A discussion needs to be held. The patient has basic education and will understand basic concepts.” (Patient 10) “It shouldn’t come through just one meeting. It comes through a lot of give and take when you begin to understand. You have to be very understanding towards the patient’s needs.” (Patient 9). “Minimally I am looking at two interactions with the doctor, and a weeklong thinking period in between by which time the patient can think about it and to her other family members.” (Public 8) “Before you accept or reject something, it takes time to make that decision. The way it's told and the way it's delivered must be good.” (Public 6) “The doctors should spend time with the patient and the family to explain the pros and cons and the probabilities. The purpose of the trial needs to be explained alongside answering the question ‘Why me?’ ‘Why you are doing the trial with me?’ And what are the probable outcomes?’’ (Public 7) “I mean detailed explanation spending more time, giving consequence or potential impact (of participating in the trial). Both positive and negative side needs to be explained. (Public 9) “We start the discussion process early, and we continue the discussion at each visit so that they know that ‘I have some treatment options to follow up’ after this treatment is over. So, the compliance remains in high, and they always visit us and over the time when we discuss multiple times different aspects of those trial interventions, and the acceptance increases.” (Health staff 10)
**Patient information materials for clinical trials**	
Language of the patient	“I think break it down, in a language that is accessible to the person concerned.” (Public 8) “Vernacularize it as much as possible. I mean, a lot of discussions that we do around issues of life, health, and consent is so circumscribed by language, because it comes from people like us.” (Public 8)
Using both audio-visual and printed patient information material	“Audio visual I think makes sense in my opinion because more than the patient, the family members also become the decision makers here. So, for them to also understand along with the patient, I think audio visual does a good job.” (Public 3) “I would say that a combination of both (leaflet and video) probably helps because once you go through the audio visual, you get some bit of an idea and then people generally tend to forget about it. The PDF leaflet would be there as a reminder of what we have seen and be a synopsis of the entire thing. I think a combination of both probably will work better.” (Public 3)
**Explaining potential benefits / adverse effects**	
Discussing pros and cons	As a patient I would appreciate it if things were explained to me individually as a person. I need to be explained the pros and cons. (patient 4) I think it is necessary for the patient’s side to know everything. (Carer 7)
Challenges of understanding quantitative benefits/risks	On being asked about the amount of risk reduction (in quantitative terms) on the potential risk of relapse with the experimental treatment perceived to be beneficial by patients, one patient replied “No, I don’t think people try to measure this. I don’t see it (the benefit) as measurable. I do not measure it with a quantitative scale” (Patient 5) “To me if it is 6% or 5% or 2% additional benefit I would likely come (to participate in the trial).” (Carer 4) “30% benefit is probably acceptable. A 10% (benefit) would make patients quite naturally think, ‘What if I am not part of it?’”. (Carer 8) “For those who are facing death, they have no other strategy but to take up anything that is offered.” (Carer 3) “So that extra 10% chance of survival, yes, that might encourage some people to go for further trials.” (Public 4)
Detailing only common potential adverse events	“I think they (patients) should be told all the side effects. The patient should know; they have a right to know.” (Patient 5) “The patient should be told just the common side effects, because if you tell them in too much details, they will not agree. They will not understand also. It is important for them to know that there are some side effects involved. Just mention the common side effects and see how they react” (Carer 10)
Fear that serious adverse effects are not mentioned	“If there is a rural town if there is a very serious adverse side effect, the chances of that news coming out to the public is very, very less. But if it happens on a western world, even in big cities of India it’s very difficult to hide from media or news.” (Public 7)
Mentioning possibility of dying (if relevant)	“Yes, absolutely, patients should definitely be informed about the possibility of death.” (Patient 5) “Even if you will live for a year more, you will at least live. This is a bit tough. Those who are strong minded will say that it’s ok. What can happen? If not today, tomorrow I will die.” (Patient 11) “If the patient comes voluntarily for this trial, it is his or her right to know all the side effects, regarding the death also, though it is rarest to rare, they still should be known to the patient.” (Carer 4) “Ethically you should inform the patient about the possibility of death, but they will be very scared to participate in the trial. But if you also mention that the chances are rare. You emphasize on the words that, ‘chances are really rare’. Otherwise, people will think that it is the only side effect.” (Carer 10) “If it is seen that one in ten thousand persons experience these side effects, I will say yes.” (Carer 2)
**Consent**	
Prerequisites for ‘consent’ – honesty, transparency, and ability to withdraw	“Two things mainly, one is the right to information, complete honesty, transparency because ultimately, even if the hospital compensates if the death happens it doesn’t bring anybody back. The ‘right to information’ and ‘transparency’ and the ‘right to back out’ is crucial. If they agree and later think that ‘this is not actually going well’ then, they should not be forced into attending it.” (Carer 7) “I would always prefer the doctor and the institution say the truth and the whole truth in case of any clinical trial.” (Public 5)
The verbal and non-verbal communication skills of person explaining the clinical trial	“Who is telling matters a lot. The body language of the person who is saying it also has an effect. If they are told in a caring way, then they will start thinking. Consent may take time, but it should make them think. Poor people are in a place where if they're made to understand nicely then they will give consent.” (Public 6)
Motivation – altruism, trust	“When you hear of a medical trial being done, you are doing it more for the greater good and for the future advancement of medicine and people would want to be part of something like that to benefit future generations.” (Carer 8)
Motivation – financial pressures, power imbalance	“In this society, which is based on the inequity of power, doctors, more often than not, are in our culture akin to God. If the patient relies on the doctor, he will consent irrespective of knowing about the consequences because the belief is that ‘doctors cannot do anything wrong, and he will do what is good for you’. This entire gamut of psychological reliance on the doctor doesn’t necessarily go all in favor of the doctor. The doctors have been put up in the pedestal of superiority, they are the one to give, we are the one to receive. As a result, so often because of the smile, because of the eye contact because of the politeness, because of even the minimal improvement in the situation, even these factors make a doctor immensely godly. And in this context, going in for a consent-based sort of educated consent is difficult.” (Public 5)
Presence of family when signing	“It is not possible to give consent on one’s own, and therefore consent can be given only after discussing the opinion of everyone in one place, then the opinion can be formed.” (Carer 2).

* the example discussed was of maintenance therapy after completion of initial cancer treatment
